# Cement extravasation resulting in L5 radiculopathy as a complication of percutaneous Sacroplasty: A case report

**DOI:** 10.1016/j.inpm.2022.100067

**Published:** 2022-02-03

**Authors:** Keith F. Polston, Micheal Murphy, Benjamin D. Westerhaus, Vinicius Tieppo Francio, Anthony Giuffrida, John Alm

**Affiliations:** a The University of Kansas Medical Center, Department of Rehabilitation Medicine, Kansas City, KS, 66160, USA; b Cantor Spine Institute, Fort Lauderdale, FL, 33306, USA

Dear Editor,

Sacral insufficiency fractures are a debilitating cause of low back pain and have been described in patients with osteoporosis, malignancy, and rheumatoid arthritis [1]. Previously described management for most cases consisted of oral analgesics, relative rest or immobilization, and physical therapy [2]. However, these conservative measures often result in limited pain reduction. Percutaneous sacroplasty, a procedure in which cement is injected into the fractured portion of the sacrum under fluoroscopy or computed tomography (CT) guidance, has emerged as a favorable treatment option to improve pain and restore stability to the fractured bone. A literature review found consistent, clinically and statistically significant, and lasting reduction in visual analog scale (VAS) pain scores following percutaneous sacroplasty in patients with sacral insufficiency fracture [3]. In the largest study analyzed in this review, average VAS pain scores in 52 patients decreased from 8.1 pre-procedure; to 3.4 immediately after procedure, to 2.5 at two, 2.1 at four, 1.7 at twelve, 1.4 at twenty-four, and 0.8 at fifty-two weeks post-procedure [4].

Cement extravasation is the most common complication of percutaneous sacroplasty, and radiculopathy is a rare additional consequence [5,6]. Mahmood et al. (2019) reported two studies with S1 radiculopathy caused by cement extravasation following sacroplasty. Furthermore, Barber et al. published an additional case report of S1 radiculopathy as sequela of cement extravasation during this procedure [7]. Chandra et al. (2019) analyzed pooled data on 861 subjects after sacroplasty and determined that the adverse event rate for radiculopathy following this procedure is 0.3% [8]. Surgical decompression has been described as a successful management option of this rare complication, such as in the case reported by Barber et al. Here, we present a case of L5 radiculopathy secondary to cement extravasation as a complication of percutaneous sacroplasty. To our knowledge, at the time of writing, this is the first reported case of lumbar radiculopathy as a sequela of cement extravasation in percutaneous sacroplasty. In addition, our approach to management differs from previously described cases in that an interdisciplinary, non-surgical approach was taken to successfully treat the patient's pain and functional deficits.

A 51-year-old female with a history of acute myeloid leukemia (AML) in remission status post bone marrow transplant one year prior to presentation, chronic graft versus host disease on chronic immunosuppression with tacrolimus and prednisone, fibromyalgia, depression, and anxiety was hospitalized for left low back, hip, and leg pain. She had recently undergone bilateral percutaneous sacroplasty for axial low back pain in the setting of bilateral sacral ala insufficiency fractures as an outpatient 3 weeks prior to presentation. Following the procedure, she developed an acute worsening in her left-sided low back pain in addition to new radiating pain from the hip to the lateral aspect of the left leg to the ankle. She also developed weakness in her left lower extremity manifested by poor toe clearance and hip flexion during swing phase of ambulation. She denied having experienced bowel or bladder incontinence, balance impairment, or sensation loss over the buttocks, perineum, or inner surface of the thighs.

At the time of initial assessment, physical exam revealed a fatigued- and ill-appearing woman who appeared older than her stated age. She had trace, distal bilateral lower extremity pitting edema. Manual muscle testing revealed 3/5 strength in the left extensor hallucis longus and 4/5 strength in the left hip flexors, although limited secondary to pain; otherwise, strength was 5/5 in all other upper and lower extremity muscles tested. Sensation was intact to light touch in all dermatomes tested throughout the bilateral upper and lower extremities. Straight leg raise and slump testing elicited left lower extremity radicular pain in the L5 distribution. Deep tendon reflexes were 2+ and symmetrical at the bilateral patellar tendons. There was no evidence of upper motor neuron signs bilaterally with no clonus at the ankles and plantarflexion of the great toe with plantar reflex testing.

This patient's symptoms of pain and weakness, beginning less than one day after undergoing percutaneous sacroplasty, lends itself to the possibility of a procedure-related complication. Given the patient's symptoms of radiating pain into the ankle with weakness of the extensor hallucis longus muscle, reported functional decline, and positive neurodynamic testing (straight leg raise and slump testing), a diagnosis of left lumbosacral radiculopathy is most likely. Nearby anatomical structures should also be considered when forming a differential diagnosis. Lumbosacral plexopathy, isolated neuropathy, vertebral compression fracture, and injury to hip flexor musculature could also be considered. In addition to a thorough history and physical exam, further diagnostic testing could include advanced imaging such as CT or magnetic resonance imaging (MRI). Furthermore, electromyography/nerve conduction study (EMG/NCS) could be considered to help localize this patient's neurological deficit. The patient ultimately underwent a CT without contrast of the lumbosacral spine and pelvis.

CT without contrast of the lumbosacral spine demonstrated a moderate amount of cement extravasation ventral to the left sacral ala, deep to the iliac vasculature and psoas muscle, with close approximation to the course of the left L5 nerve root (Fig. 1). The patient's CT results were consistent with clinical suspicion, and she was diagnosed with a left L5 radiculopathy secondary to cement extravasation. Additionally, the cement appears to contact the posterior aspect of the iliopsoas muscle, which likely led to inflammation and could explain the patient's pain and weakness with hip flexion (Fig. 2). Orthopedic surgery was consulted to assess for potential operative intervention but felt that surgery would be too high-risk and recommended conservative management with a trial of gabapentin and an L5-S1 transforaminal epidural steroid injection.

**Fig. 1:**
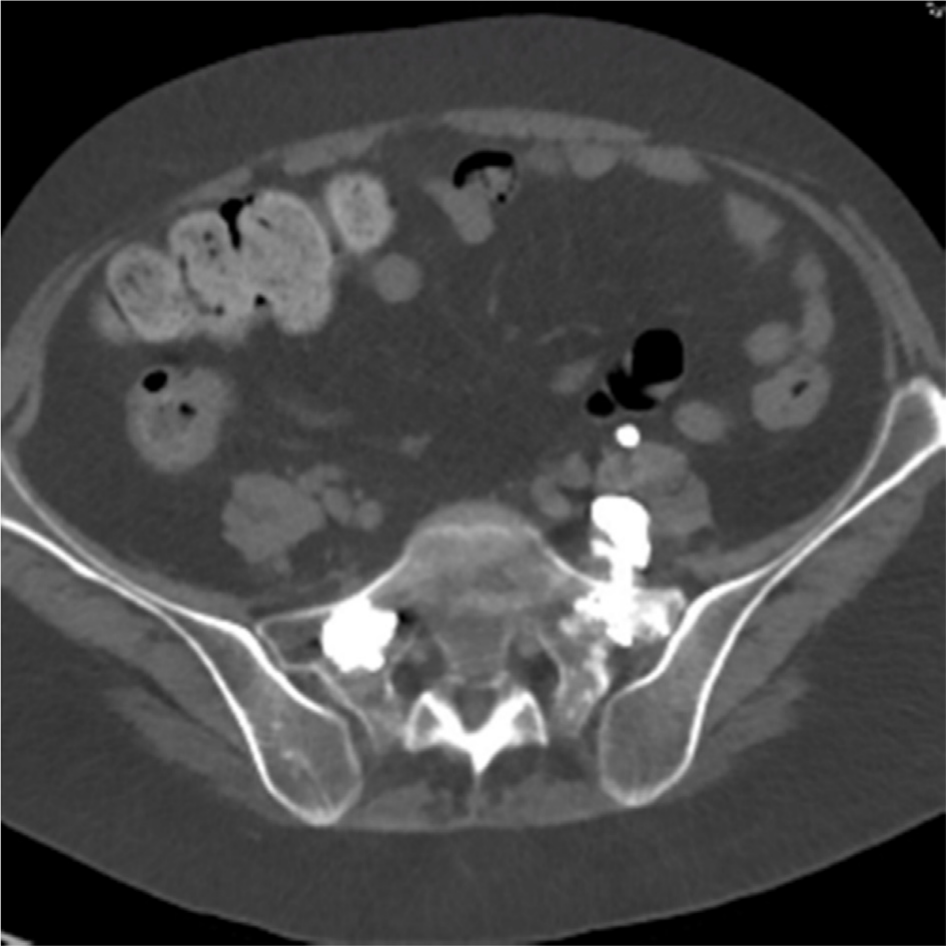
Axial view CT without contrast of lumbosacral spine demonstrating cement extravasation anterior to left sacral ala along the course of left L5 nerve root.

**Fig. 2:**
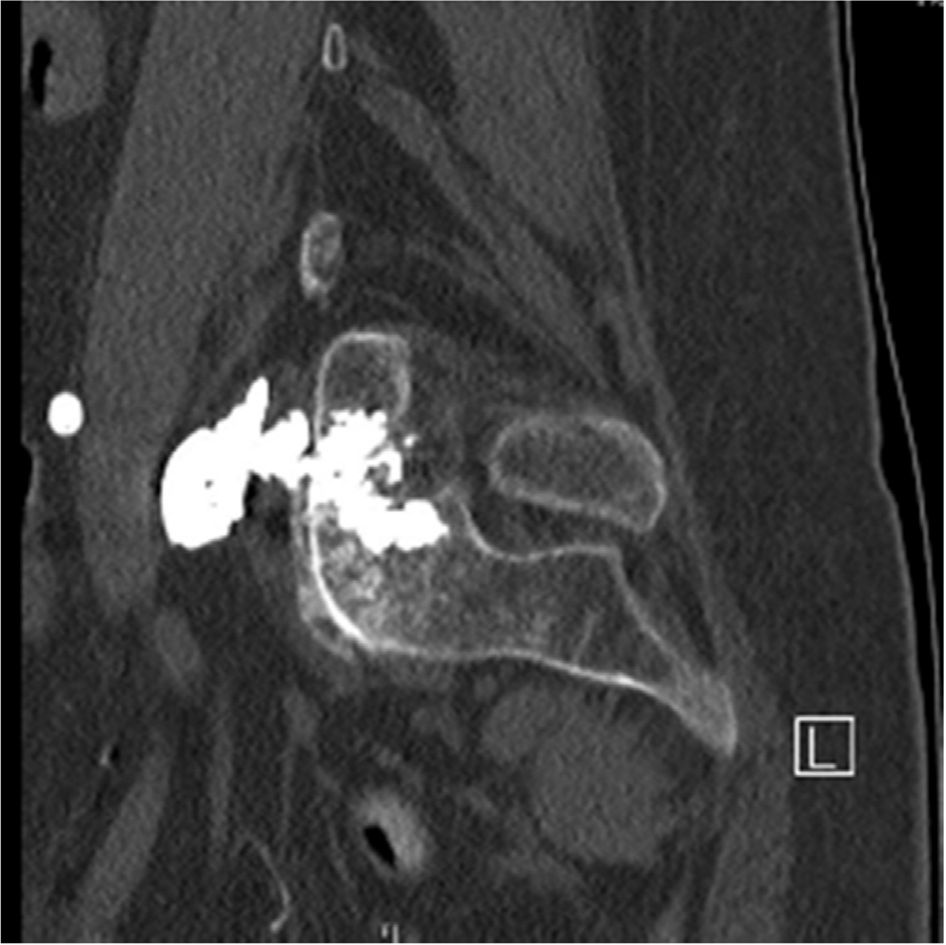
Sagittal view CT without contrast of lumbosacral spine demonstrating cement extravasation extending from the sacral ala posteriorly and abutting the iliopsoas muscle anteriorly.

The patient received these interventions and experienced moderate pain relief with a VAS pain score reduction from 8 to 5. Gabapentin was ultimately discontinued due to the development of altered mental status, and she was subsequently admitted to inpatient rehabilitation to address her functional deficits. During her rehabilitation course, her pain was effectively controlled with acetaminophen along with long-acting and short-acting opioids as needed. She demonstrated significant improvement from minimum assist to independent with a four-wheeled walker for ambulation, transfers, and toileting by the time of discharge. Ultimately, she was discharged home with home health physical therapy and occupational therapy in addition to having scheduled follow-up appointments with orthopedic surgery, pain management, rehabilitation medicine, and primary care. She reported 50% pain reduction at her follow-up appointment with pain management and was scheduled for further follow-up. Furthermore, her left hip flexion weakness had resolved, and she maintained functional improvement such that she was able to ambulate independently.

Sacral insufficiency fractures are painful and debilitating injuries that may lead to spinal abnormalities, impaired mobility, and limited function, with patients consequently at high risk of developing complications such as venous thrombosis, pulmonary embolism, pressure injuries, deformities, and chronic pain, particularly in the elderly [3]. Incidence has been estimated to affect 1–5% of high-risk elderly, such as postmenopausal woman, immunocompromised, and osteoporotic patients [2]. These fractures and their complications can be difficult to diagnose, often requiring advanced clinical skills and diagnostic imaging, and as a result, diagnosis may be delayed for many weeks. This factor is important because early diagnosis and timely intervention are essential to preserve mobility and avoid deconditioning and immobility [[2], [3], [4]]. Percutaneous sacroplasty is a procedure in which cement is injected into the fractured portion of the sacrum under fluoroscopy or computed tomography (CT) guidance, aiming to reduce pain and facilitate early mobilization. Although percutaneous sacroplasty has shown these benefits, there are significant potential complications. A systematic review of complications found cement extravasation to be the most common [5]. In rare instances, cement extravasation has been reported to result in S1 radiculopathy via contact with and thermal injury to nearby nerve roots [6]. Polymethyl methacrylate (PMMA) cement is often used in this procedure and is known to undergo an exothermic reaction leading to heat transfer to nearby structures [9]. In this case report, the patient was diagnosed with a left L5 radiculopathy secondary to cement extravasation eliciting an inflammatory thermal reaction along the L5 nerve root and likely contacting the posterior aspect of the iliopsoas muscle (Fig. 2), which could explain the patient's pain and weakness with hip flexion.

To our knowledge, this is the only case in the literature reporting cement extravasation leading to lumbar (L5) radiculopathy that was treated non-operatively. Most cases reported in the literature are of sacral (S1) radiculopathy and are often managed surgically [5,7]. The management approach described by Barber et al. (2013) differed from the present case in that the patient was successfully treated with surgical decompression [7]. In the present case, the consulting surgeon felt that an attempt to remove the cement would require an anterior retroperitoneal approach to the spine and place the L5 nerve root at risk for further injury. Furthermore, the patient's medical comorbidities including AML and chronic immunosuppression heightened the risk of potential surgical complications including infection and poor wound healing. The overall approach demonstrated a favorable patient-centered response to a more conservative, multi-faceted approach including oral analgesia, an epidural steroid injection, inpatient rehabilitation, home health physical and occupational therapy, and close follow-up care with pain management.

In the present case, establishing a clear diagnosis was challenging for several reasons. First, the patient's pre-existing chronic pain in the setting of sacral insufficiency fractures made it difficult to decipher which aspects of her pain were acute versus chronic. Second, the nature of the cement extravasation was such that it contacted multiple structures. The cement's proximity to the iliopsoas muscle, coupled with the patient's weakness and pain with hip flexion, made alternative diagnoses such as femoral neuropathy plausible.

While the diagnostic test of choice in the present case was a CT scan, an argument could have been made for other diagnostic tests including MRI or electrodiagnostic testing. In this case, MRI was not able to be performed due to implanted device incompatibility in this patient. Furthermore, thrombocytopenia at the time of presentation made electrodiagnostic testing risky. In general, MRI is the preferred modality in suitable patients who are suspected of having radiculopathy due to its greater sensitivity and superior demonstration of soft tissue [10]. However, CT proved sufficient in this case in demonstrating the patient's bony abnormalities, including the cement extravasation. Should the patient's pain and functional deficits have been refractory to treatment, or if alternative diagnoses were likely, electrodiagnostic testing would have been reasonable to consider next.

Percutaneous sacroplasty is a generally safe and efficacious procedure that results in improved pain for most patients with sacral insufficiency fractures [5]. As is true of any procedure, complications are possible, and it is important to recognize potential risks. While the most common complication is cement extravasation, it can rarely lead to radiculopathy. In this complex case, the patient required a multidisciplinary approach that included inpatient acute pain service, interventional chronic pain management, orthopedic surgery, oncology, and physical medicine and rehabilitation. In addition to emphasizing a multi-specialty approach to diagnosis and treatment, we highlight the importance of post-acute care needs, including a continuum of care with rehabilitation medicine to address impairments in function and debilitating pain in this patient.

## Financial disclosures

None.

## Declaration of competing interest

The authors declare no conflicts of interest.

## References

[1] Weber M., Hasler P., Gerber H. (1993 Dec). Insufficiency fractures of the sacrum. Twenty cases and review of the literature. Spine.

[2] Tsiridis E., Upadhyay N., Giannoudis P.V. (2006 Dec). Sacral insufficiency fractures: current concepts of management. Osteoporos Int.

[3] Gupta A.C., Yoo A.J., Stone J., Barr J.C., Brook A., Tutton S., Ortiz O., Hirsch A.E., Larvie M., Frey M.E., Jayaraman M.V., Hirsch J.A. (2012 Sep). Percutaneous sacroplasty. J Neurointerventional Surg.

[4] Frey M.E., Depalma M.J., Cifu D.X., Bhagia S.M., Carne W., Daitch J.S. (2008 Mar-Apr). Percutaneous sacroplasty for osteoporotic sacral insufficiency fractures: a prospective, multicenter, observational pilot study. Spine J.

[5] Mahmood B., Pasternack J., Razi A., Saleh A. (2019 Sep). Safety and efficacy of percutaneous sacroplasty for treatment of sacral insufficiency fractures: a systematic review. J Spine Surg.

[6] Bastian J.D., Keel M.J., Heini P.F., Seidel U., Benneker L.M. (2012 Feb). Complications related to cement leakage in sacroplasty. Acta Orthop Belg.

[7] Barber S.M., Livingston A.D., Cech D.A. (2013 May). Sacral radiculopathy due to cement leakage from percutaneous sacroplasty, successfully treated with surgical decompression. J Neurosurg Spine.

[8] Chandra V., Wajswol E., Shukla P., Contractor S., Kumar A. (2019 Nov). Safety and efficacy of sacroplasty for sacral fractures: a systematic review and meta-analysis. J Vasc Intervent Radiol.

[9] Gheduzzi S., Webb J.J., Miles A.W. (2006). Mechanical characterization of three percutaneous vertebroplasty biomaterials. J Mater Sci Mater Med.

[10] Rao D., Scuderi G., Scuderi C., Grewal R., Sandhu S.J. (2018). The use of imaging in management of patients with low back pain. J Clin Imaging Sci.

